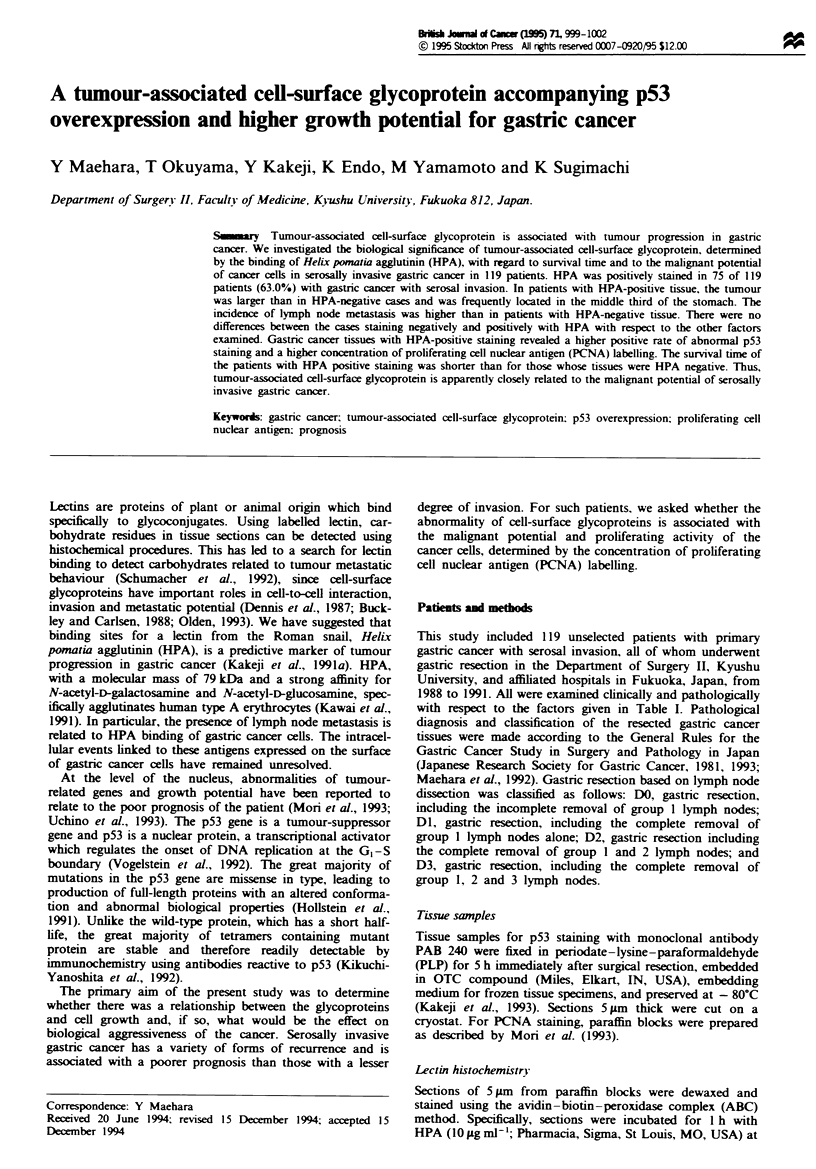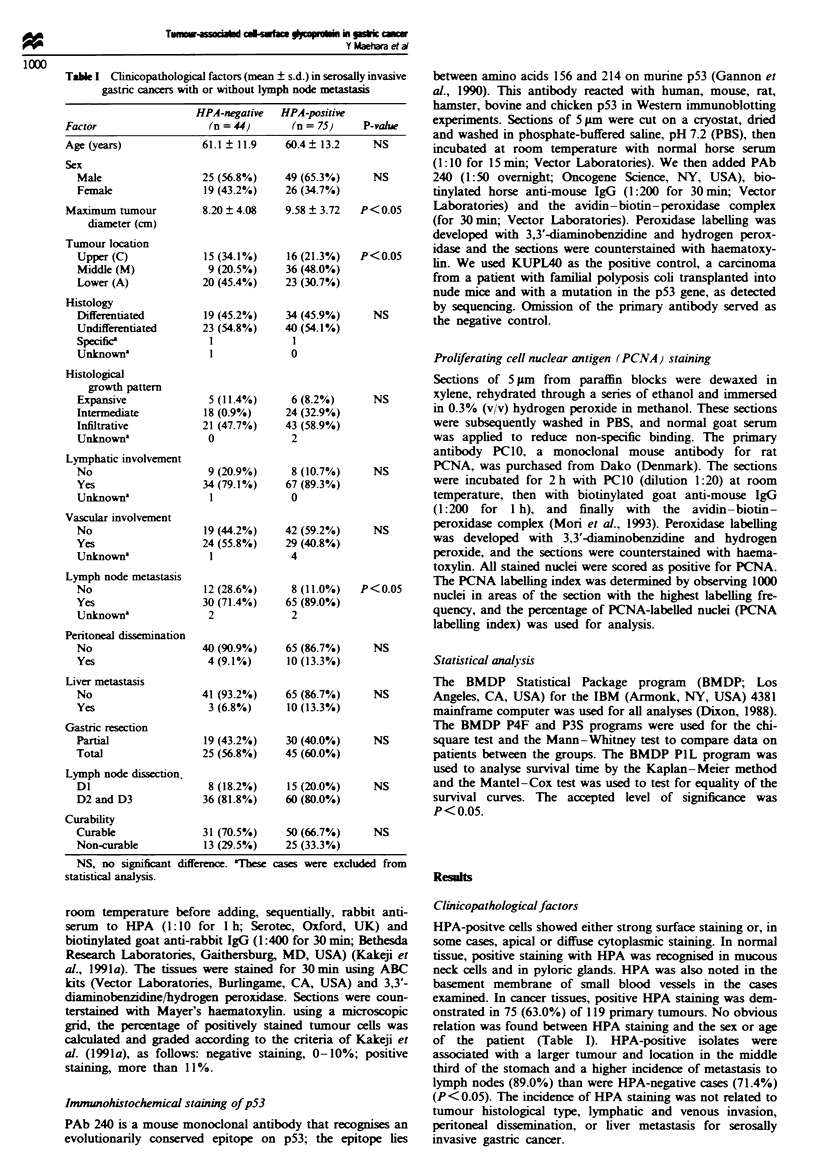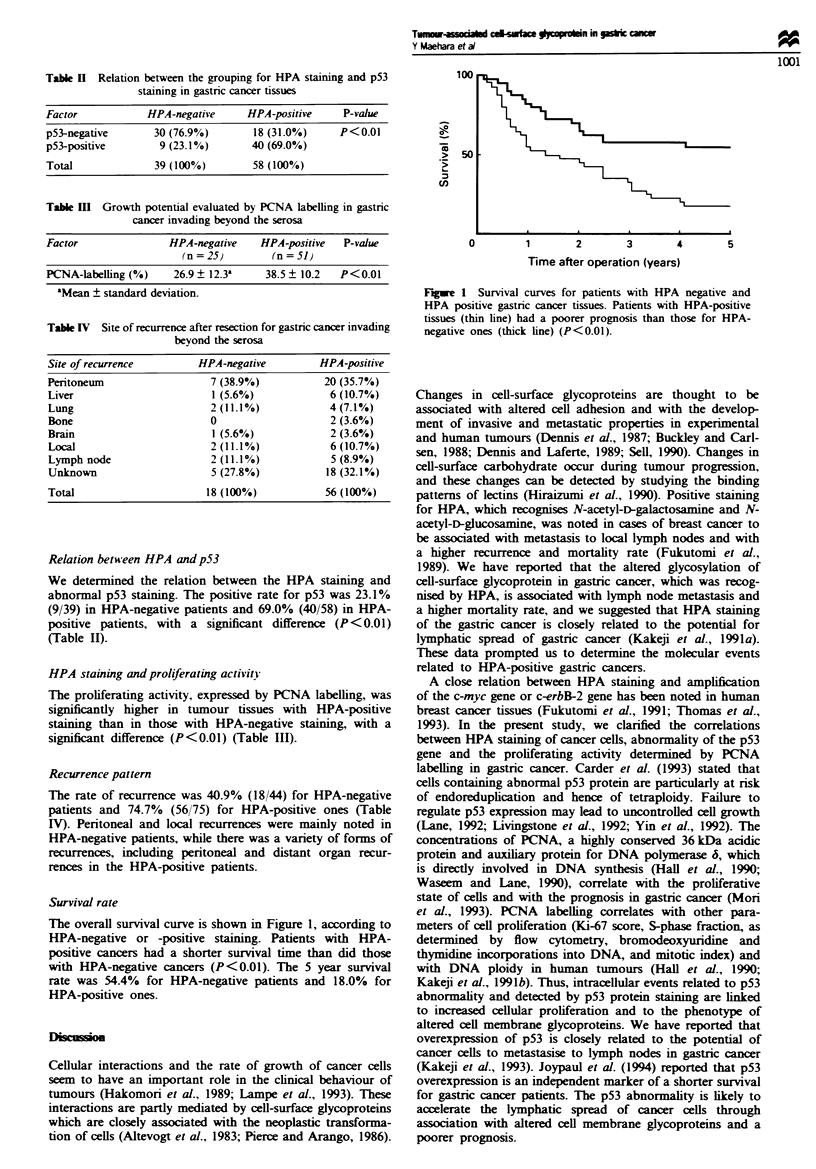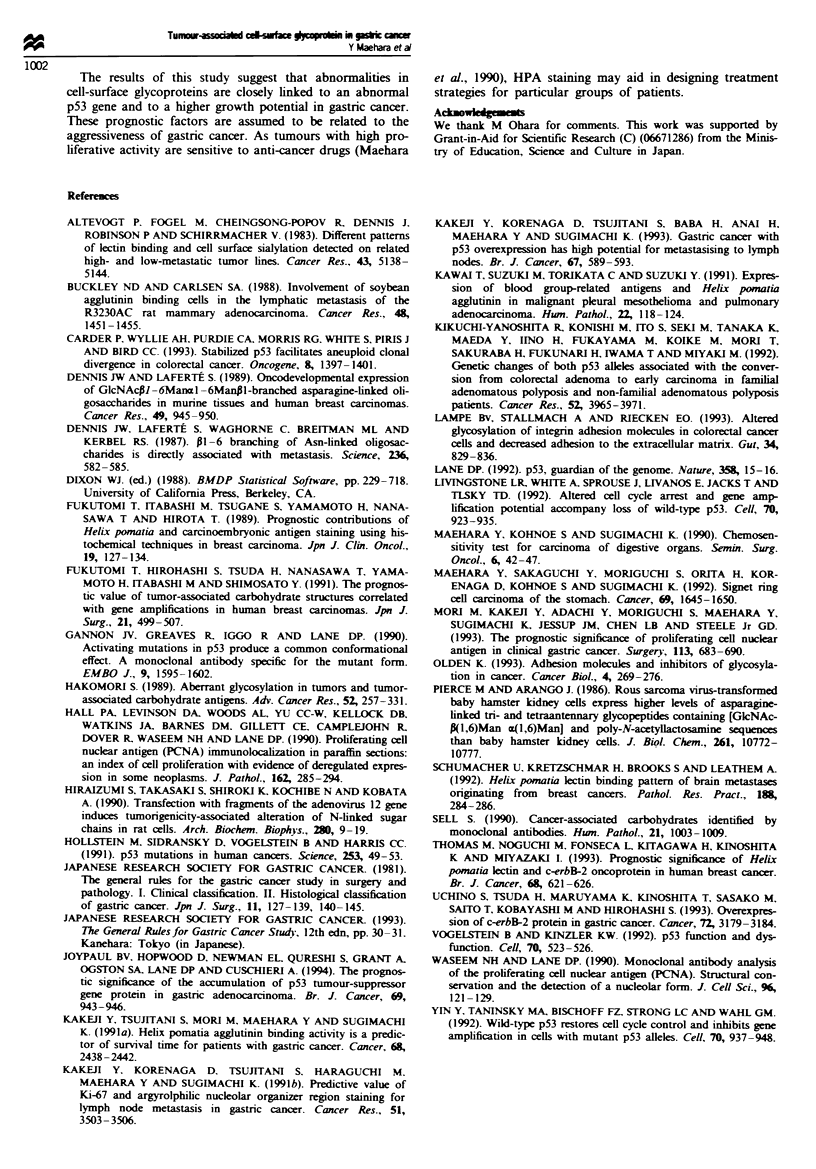# A tumour-associated cell-surface glycoprotein accompanying p53 overexpression and higher growth potential for gastric cancer.

**DOI:** 10.1038/bjc.1995.192

**Published:** 1995-05

**Authors:** Y. Maehara, T. Okuyama, Y. Kakeji, K. Endo, M. Yamamoto, K. Sugimachi

**Affiliations:** Department of Surgery II, Faculty of Medicine, Kyushu University, Fukuoka, Japan.

## Abstract

Tumour-associated cell-surface glycoprotein is associated with tumour progression in gastric cancer. We investigated the biological significance of tumour-associated cell-surface glycoprotein, determined by the binding of Helix pomatia agglutinin (HPA), with regard to survival time and to the malignant potential of cancer cells in serosally invasive gastric cancer in 119 patients. HPA was positively stained in 75 of 119 patients (63.0%) with gastric cancer with serosal invasion. In patients with HPA-positive tissue, the tumour was larger than in HPA-negative cases and was frequently located in the middle third of the stomach. The incidence of lymph node metastasis was higher than in patients with HPA-negative tissue. There were no differences between the cases staining negatively and positively with HPA with respect to the other factors examined. Gastric cancer tissues with HPA-positive staining revealed a higher positive rate of abnormal p53 staining and a higher concentration of proliferating cell nuclear antigen (PCNA) labelling. The survival time of the patients with HPA positive staining was shorter than for those whose tissues were HPA negative. Thus, tumour-associated cell-surface glycoprotein is apparently closely related to the malignant potential of serosally invasive gastric cancer.


British Journal of Cancer (1995) 71, 999-1002

© 1995 Stockton Press All rights reserved 0007-0920/95 $12.00

### A tumour-associated cell-surface glycoprotein accompanying p53 overexpression and higher growth potential for gastric cancer

Y Maehara, T Okuyama, Y Kakeji, K Endo, M Yamamoto and K Sugimachi

*Department of Surgery II, Faculty of Medicine, Kyushu University, Fukuoka 812, Japan.*

**Summary** Tumour-associated cell-surface glycoprotein is associated with tumour progression in gastric cancer. We investigated the biological significance of tumour-associated cell-surface glycoprotein, determined by the binding of *Helix pomatia* agglutinin (HPA), with regard to survival time and to the malignant potential of cancer cells in serosally invasive gastric cancer in 119 patients. HPA was positively stained in 75 of 119 patients (63.0%) with gastric cancer with serosal invasion. In patients with HPA-positive tissue, the tumour was larger than in HPA-negative cases and was frequently located in the middle third of the stomach. The incidence of lymph node metastasis was higher than in patients with HPA-negative tissue. There were no differences between the cases staining negatively and positively with HPA with respect to the other factors examined. Gastric cancer tissues with HPA-positive staining revealed a higher positive rate of abnormal p53 staining and a higher concentration of proliferating cell nuclear antigen (PCNA) labelling. The survival time of the patients with HPA positive staining was shorter than for those whose tissues were HPA negative. Thus, tumour-associated cell-surface glycoprotein is apparently closely related to the malignant potential of serosally invasive gastric cancer.

**Keywords**: gastric cancer; tumour-associated cell-surface glycoprotein; p53 overexpression; proliferating cell nuclear antigen; prognosis

Lectins are proteins of plant or animal origin which bind specifically to glycoconjugates. Using labelled lectin, carbohydrate residues in tissue sections can be detected using histochemical procedures. This has led to a search for lectin binding to detect carbohydrates related to tumour metastatic behaviour (Schumacher *et al.*, 1992), since cell-surface glycoproteins have important roles in cell-to-cell interaction, invasion and metastatic potential (Dennis *et al.*, 1987; Buckley and Carlsen, 1988; Olden, 1993). We have suggested that binding sites for a lectin from the Roman snail, *Helix pomatia* agglutinin (HPA), is a predictive marker of tumour progression in gastric cancer (Kakeji *et al.*, 1991*a*). HPA, with a molecular mass of 79 kDa and a strong affinity for *N*-acetyl-D-galactosamine and *N*-acetyl-D-glucosamine, specifically agglutinates human type A erythrocytes (Kawai *et al.*, 1991). In particular, the presence of lymph node metastasis is related to HPA binding of gastric cancer cells. The intracellular events linked to these antigens expressed on the surface of gastric cancer cells have remained unresolved.

At the level of the nucleus, abnormalities of tumour-related genes and growth potential have been reported to relate to the poor prognosis of the patient (Mori *et al.*, 1993; Uchino *et al.*, 1993). The p53 gene is a tumour-suppressor gene and p53 is a nuclear protein, a transcriptional activator which regulates the onset of DNA replication at the $G_1$-S boundary (Vogelstein *et al.*, 1992). The great majority of mutations in the p53 gene are missense in type, leading to production of full-length proteins with an altered conformation and abnormal biological properties (Hollstein *et al.*, 1991). Unlike the wild-type protein, which has a short half-life, the great majority of tetramers containing mutant protein are stable and therefore readily detectable by immunochemistry using antibodies reactive to p53 (Kikuchi-Yanoshita *et al.*, 1992).

The primary aim of the present study was to determine whether there was a relationship between the glycoproteins and cell growth and, if so, what would be the effect on biological aggressiveness of the cancer. Serosally invasive gastric cancer has a variety of forms of recurrence and is associated with a poorer prognosis than those with a lesser degree of invasion. For such patients, we asked whether the abnormality of cell-surface glycoproteins is associated with the malignant potential and proliferating activity of the cancer cells, determined by the concentration of proliferating cell nuclear antigen (PCNA) labelling.

### Patients and methods

This study included 119 unselected patients with primary gastric cancer with serosal invasion, all of whom underwent gastric resection in the Department of Surgery II, Kyushu University, and affiliated hospitals in Fukuoka, Japan, from 1988 to 1991. All were examined clinically and pathologically with respect to the factors given in Table I. Pathological diagnosis and classification of the resected gastric cancer tissues were made according to the General Rules for the Gastric Cancer Study in Surgery and Pathology in Japan (Japanese Research Society for Gastric Cancer, 1981, 1993; Maehara *et al.*, 1992). Gastric resection based on lymph node dissection was classified as follows: D0, gastric resection, including the incomplete removal of group 1 lymph nodes; D1, gastric resection, including the complete removal of group 1 lymph nodes alone; D2, gastric resection including the complete removal of group 1 and 2 lymph nodes; and D3, gastric resection, including the complete removal of group 1, 2 and 3 lymph nodes.

### *Tissue samples*

Tissue samples for p53 staining with monoclonal antibody PAB 240 were fixed in periodate-lysine-paraformaldehyde (PLP) for 5 h immediately after surgical resection, embedded in OTC compound (Miles, Elkart, IN, USA), embedding medium for frozen tissue specimens, and preserved at − 80°C (Kakeji *et al.*, 1993). Sections 5 μm thick were cut on a cryostat. For PCNA staining, paraffin blocks were prepared as described by Mori *et al.* (1993).

### *Lectin histochemistry*

Sections of 5 μm from paraffin blocks were dewaxed and stained using the avidin-biotin-peroxidase complex (ABC) method. Specifically, sections were incubated for 1 h with HPA (10 μg ml$^{-1}$; Pharmacia, Sigma, St Louis, MO, USA) at

Correspondence: Y Maehara
Received 20 June 1994; revised 15 December 1994; accepted 15 December 1994

**Table I** Clinicopathological factors (mean ± s.d.) in serosally invasive gastric cancers with or without lymph node metastasis

| *Factor* | *HPA-negative* (n = 44) | *HPA-positive* (n = 75) | P-*value* |
|---|---|---|---|
| Age (years) | 61.1 ± 11.9 | 60.4 ± 13.2 | NS |
| Sex | | | |
| Male | 25 (56.8%) | 49 (65.3%) | NS |
| Female | 19 (43.2%) | 26 (34.7%) | |
| Maximum tumour diameter (cm) | 8.20 ± 4.08 | 9.58 ± 3.72 | $P<0.05$ |
| Tumour location | | | |
| Upper (C) | 15 (34.1%) | 16 (21.3%) | $P<0.05$ |
| Middle (M) | 9 (20.5%) | 36 (48.0%) | |
| Lower (A) | 20 (45.4%) | 23 (30.7%) | |
| Histology | | | |
| Differentiated | 19 (45.2%) | 34 (45.9%) | NS |
| Undifferentiated | 23 (54.8%) | 40 (54.1%) | |
| Specific[a] | 1 | 1 | |
| Unknown[a] | 1 | 0 | |
| Histological growth pattern | | | |
| Expansive | 5 (11.4%) | 6 (8.2%) | NS |
| Intermediate | 18 (0.9%) | 24 (32.9%) | |
| Infiltrative | 21 (47.7%) | 43 (58.9%) | |
| Unknown[a] | 0 | 2 | |
| Lymphatic involvement | | | |
| No | 9 (20.9%) | 8 (10.7%) | NS |
| Yes | 34 (79.1%) | 67 (89.3%) | |
| Unknown[a] | 1 | 0 | |
| Vascular involvement | | | |
| No | 19 (44.2%) | 42 (59.2%) | NS |
| Yes | 24 (55.8%) | 29 (40.8%) | |
| Unknown[a] | 1 | 4 | |
| Lymph node metastasis | | | |
| No | 12 (28.6%) | 8 (11.0%) | $P<0.05$ |
| Yes | 30 (71.4%) | 65 (89.0%) | |
| Unknown[a] | 2 | 2 | |
| Peritoneal dissemination | | | |
| No | 40 (90.9%) | 65 (86.7%) | NS |
| Yes | 4 (9.1%) | 10 (13.3%) | |
| Liver metastasis | | | |
| No | 41 (93.2%) | 65 (86.7%) | NS |
| Yes | 3 (6.8%) | 10 (13.3%) | |
| Gastric resection | | | |
| Partial | 19 (43.2%) | 30 (40.0%) | NS |
| Total | 25 (56.8%) | 45 (60.0%) | |
| Lymph node dissection | | | |
| D1 | 8 (18.2%) | 15 (20.0%) | NS |
| D2 and D3 | 36 (81.8%) | 60 (80.0%) | |
| Curability | | | |
| Curable | 31 (70.5%) | 50 (66.7%) | NS |
| Non-curable | 13 (29.5%) | 25 (33.3%) | |

NS, no significant difference. [a]These cases were excluded from statistical analysis.

room temperature before adding, sequentially, rabbit antiserum to HPA (1:10 for 1 h; Serotec, Oxford, UK) and biotinylated goat anti-rabbit IgG (1:400 for 30 min; Bethesda Research Laboratories, Gaithersburg, MD, USA) (Kakeji *et al.*, 1991*a*). The tissues were stained for 30 min using ABC kits (Vector Laboratories, Burlingame, CA, USA) and 3,3'-diaminobenzidine/hydrogen peroxidase. Sections were counterstained with Mayer's haematoxylin. using a microscopic grid, the percentage of positively stained tumour cells was calculated and graded according to the criteria of Kakeji *et al.* (1991*a*), as follows: negative staining, 0–10%; positive staining, more than 11%.

### *Immunohistochemical staining of p53*

PAb 240 is a mouse monoclonal antibody that recognises an evolutionarily conserved epitope on p53; the epitope lies between amino acids 156 and 214 on murine p53 (Gannon *et al.*, 1990). This antibody reacted with human, mouse, rat, hamster, bovine and chicken p53 in Western immunoblotting experiments. Sections of 5 µm were cut on a cryostat, dried and washed in phosphate-buffered saline, pH 7.2 (PBS), then incubated at room temperature with normal horse serum (1:10 for 15 min; Vector Laboratories). We then added PAb 240 (1:50 overnight; Oncogene Science, NY, USA), biotinylated horse anti-mouse IgG (1:200 for 30 min; Vector Laboratories) and the avidin–biotin–peroxidase complex (for 30 min; Vector Laboratories). Peroxidase labelling was developed with 3,3'-diaminobenzidine and hydrogen peroxidase and the sections were counterstained with haematoxylin. We used KUPL40 as the positive control, a carcinoma from a patient with familial polyposis coli transplanted into nude mice and with a mutation in the p53 gene, as detected by sequencing. Omission of the primary antibody served as the negative control.

### *Proliferating cell nuclear antigen (PCNA) staining*

Sections of 5 µm from paraffin blocks were dewaxed in xylene, rehydrated through a series of ethanol and immersed in 0.3% (v/v) hydrogen peroxide in methanol. These sections were subsequently washed in PBS, and normal goat serum was applied to reduce non-specific binding. The primary antibody PC10, a monoclonal mouse antibody for rat PCNA, was purchased from Dako (Denmark). The sections were incubated for 2 h with PC10 (dilution 1:20) at room temperature, then with biotinylated goat anti-mouse IgG (1:200 for 1 h), and finally with the avidin–biotin–peroxidase complex (Mori *et al.*, 1993). Peroxidase labelling was developed with 3,3'-diaminobenzidine and hydrogen peroxide, and the sections were counterstained with haematoxylin. All stained nuclei were scored as positive for PCNA. The PCNA labelling index was determined by observing 1000 nuclei in areas of the section with the highest labelling frequency, and the percentage of PCNA-labelled nuclei (PCNA labelling index) was used for analysis.

### *Statistical analysis*

The BMDP Statistical Package program (BMDP; Los Angeles, CA, USA) for the IBM (Armonk, NY, USA) 4381 mainframe computer was used for all analyses (Dixon, 1988). The BMDP P4F and P3S programs were used for the chi-square test and the Mann–Whitney test to compare data on patients between the groups. The BMDP P1L program was used to analyse survival time by the Kaplan–Meier method and the Mantel–Cox test was used to test for equality of the survival curves. The accepted level of significance was $P<0.05$.

### Results

### *Clinicopathological factors*

HPA-positve cells showed either strong surface staining or, in some cases, apical or diffuse cytoplasmic staining. In normal tissue, positive staining with HPA was recognised in mucous neck cells and in pyloric glands. HPA was also noted in the basement membrane of small blood vessels in the cases examined. In cancer tissues, positive HPA staining was demonstrated in 75 (63.0%) of 119 primary tumours. No obvious relation was found between HPA staining and the sex or age of the patient (Table I). HPA-positive isolates were associated with a larger tumour and location in the middle third of the stomach and a higher incidence of metastasis to lymph nodes (89.0%) than were HPA-negative cases (71.4%) ($P<0.05$). The incidence of HPA staining was not related to tumour histological type, lymphatic and venous invasion, peritoneal dissemination, or liver metastasis for serosally invasive gastric cancer.

**Table II** Relation between the grouping for HPA staining and p53 staining in gastric cancer tissues

| *Factor* | *HPA-negative* | *HPA-positive* | P-*value* |
|---|---|---|---|
| p53-negative | 30 (76.9%) | 18 (31.0%) | $P<0.01$ |
| p53-positive | 9 (23.1%) | 40 (69.0%) | |
| Total | 39 (100%) | 58 (100%) | |

**Table III** Growth potential evaluated by PCNA labelling in gastric cancer invading beyond the serosa

| *Factor* | *HPA-negative* (n = 25) | *HPA-positive* (n = 51) | P-*value* |
|---|---|---|---|
| PCNA-labelling (%) | 26.9 ± 12.3[a] | 38.5 ± 10.2 | $P<0.01$ |

[a]Mean ± standard deviation.

**Table IV** Site of recurrence after resection for gastric cancer invading beyond the serosa

| *Site of recurrence* | *HPA-negative* | *HPA-positive* |
|---|---|---|
| Peritoneum | 7 (38.9%) | 20 (35.7%) |
| Liver | 1 (5.6%) | 6 (10.7%) |
| Lung | 2 (11.1%) | 4 (7.1%) |
| Bone | 0 | 2 (3.6%) |
| Brain | 1 (5.6%) | 2 (3.6%) |
| Local | 2 (11.1%) | 6 (10.7%) |
| Lymph node | 2 (11.1%) | 5 (8.9%) |
| Unknown | 5 (27.8%) | 18 (32.1%) |
| Total | 18 (100%) | 56 (100%) |

### *Relation between HPA and p53*

We determined the relation between the HPA staining and abnormal p53 staining. The positive rate for p53 was 23.1% (9/39) in HPA-negative patients and 69.0% (40/58) in HPA-positive patients, with a significant difference ($P<0.01$) (Table II).

### *HPA staining and proliferating activity*

The proliferating activity, expressed by PCNA labelling, was significantly higher in tumour tissues with HPA-positive staining than in those with HPA-negative staining, with a significant difference ($P<0.01$) (Table III).

### *Recurrence pattern*

The rate of recurrence was 40.9% (18/44) for HPA-negative patients and 74.7% (56/75) for HPA-positive ones (Table IV). Peritoneal and local recurrences were mainly noted in HPA-negative patients, while there was a variety of forms of recurrences, including peritoneal and distant organ recurrences in the HPA-positive patients.

### *Survival rate*

The overall survival curve is shown in Figure 1, according to HPA-negative or -positive staining. Patients with HPA-positive cancers had a shorter survival time than did those with HPA-negative cancers ($P<0.01$). The 5 year survival rate was 54.4% for HPA-negative patients and 18.0% for HPA-positive ones.

### Discussion

Cellular interactions and the rate of growth of cancer cells seem to have an important role in the clinical behaviour of tumours (Hakomori *et al.*, 1989; Lampe *et al.*, 1993). These interactions are partly mediated by cell-surface glycoproteins which are closely associated with the neoplastic transformation of cells (Altevogt *et al.*, 1983; Pierce and Arango, 1986).

**Figure 1** Survival curves for patients with HPA negative and HPA positive gastric cancer tissues. Patients with HPA-positive tissues (thin line) had a poorer prognosis than those for HPA-negative ones (thick line) ($P<0.01$).

Changes in cell-surface glycoproteins are thought to be associated with altered cell adhesion and with the development of invasive and metastatic properties in experimental and human tumours (Dennis *et al.*, 1987; Buckley and Carlsen, 1988; Dennis and Laferte, 1989; Sell, 1990). Changes in cell-surface carbohydrate occur during tumour progression, and these changes can be detected by studying the binding patterns of lectins (Hiraizumi *et al.*, 1990). Positive staining for HPA, which recognises *N*-acetyl-D-galactosamine and *N*-acetyl-D-glucosamine, was noted in cases of breast cancer to be associated with metastasis to local lymph nodes and with a higher recurrence and mortality rate (Fukutomi *et al.*, 1989). We have reported that the altered glycosylation of cell-surface glycoprotein in gastric cancer, which was recognised by HPA, is associated with lymph node metastasis and a higher mortality rate, and we suggested that HPA staining of the gastric cancer is closely related to the potential for lymphatic spread of gastric cancer (Kakeji *et al.*, 1991*a*). These data prompted us to determine the molecular events related to HPA-positive gastric cancers.

A close relation between HPA staining and amplification of the c-*myc* gene or c-*erb*B-2 gene has been noted in human breast cancer tissues (Fukutomi *et al.*, 1991; Thomas *et al.*, 1993). In the present study, we clarified the correlations between HPA staining of cancer cells, abnormality of the p53 gene and the proliferating activity determined by PCNA labelling in gastric cancer. Carder *et al.* (1993) stated that cells containing abnormal p53 protein are particularly at risk of endoreduplication and hence of tetraploidy. Failure to regulate p53 expression may lead to uncontrolled cell growth (Lane, 1992; Livingstone *et al.*, 1992; Yin *et al.*, 1992). The concentrations of PCNA, a highly conserved 36 kDa acidic protein and auxiliary protein for DNA polymerase $\delta$, which is directly involved in DNA synthesis (Hall *et al.*, 1990; Waseem and Lane, 1990), correlate with the proliferative state of cells and with the prognosis in gastric cancer (Mori *et al.*, 1993). PCNA labelling correlates with other parameters of cell proliferation (Ki-67 score, S-phase fraction, as determined by flow cytometry, bromodeoxyuridine and thymidine incorporations into DNA, and mitotic index) and with DNA ploidy in human tumours (Hall *et al.*, 1990; Kakeji *et al.*, 1991*b*). Thus, intracellular events related to p53 abnormality and detected by p53 protein staining are linked to increased cellular proliferation and to the phenotype of altered cell membrane glycoproteins. We have reported that overexpression of p53 is closely related to the potential of cancer cells to metastasise to lymph nodes in gastric cancer (Kakeji *et al.*, 1993). Joypaul *et al.* (1994) reported that p53 overexpression is an independent marker of a shorter survival for gastric cancer patients. The p53 abnormality is likely to accelerate the lymphatic spread of cancer cells through association with altered cell membrane glycoproteins and a poorer prognosis.

The results of this study suggest that abnormalities in cell-surface glycoproteins are closely linked to an abnormal p53 gene and to a higher growth potential in gastric cancer. These prognostic factors are assumed to be related to the aggressiveness of gastric cancer. As tumours with high proliferative activity are sensitive to anti-cancer drugs (Maehara *et al.*, 1990), HPA staining may aid in designing treatment strategies for particular groups of patients.

**Acknowledgements**
We thank M Ohara for comments. This work was supported by Grant-in-Aid for Scientific Research (C) (06671286) from the Ministry of Education, Science and Culture in Japan.

**References**

ALTEVOGT P, FOGEL M, CHEINGSONG-POPOV R, DENNIS J, ROBINSON P AND SCHIRRMACHER V. (1983). Different patterns of lectin binding and cell surface sialylation detected on related high- and low-metastatic tumor lines. *Cancer Res.*, **43**, 5138–5144.

BUCKLEY ND AND CARLSEN SA. (1988). Involvement of soybean agglutinin binding cells in the lymphatic metastasis of the R3230AC rat mammary adenocarcinoma. *Cancer Res.*, **48**, 1451–1455.

CARDER P, WYLLIE AH, PURDIE CA, MORRIS RG, WHITE S, PIRIS J AND BIRD CC. (1993). Stabilized p53 facilitates aneuploid clonal divergence in colorectal cancer. *Oncogene*, **8**, 1397–1401.

DENNIS JW AND LAFERTÉ S. (1989). Oncodevelopmental expression of GlcNAc*β1–6*Manα1–6Man*β*1-branched asparagine-linked oligosaccharides in murine tissues and human breast carcinomas. *Cancer Res.*, **49**, 945–950.

DENNIS JW, LAFERTÉ S, WAGHORNE C, BREITMAN ML AND KERBEL RS. (1987). *β*1–6 branching of Asn-linked oligosaccharides is directly associated with metastasis. *Science*, **236**, 582–585.

DIXON WJ. (ed.) (1988). *BMDP Statistical Software*, pp. 229–718. University of California Press, Berkeley, CA.

FUKUTOMI T, ITABASHI M, TSUGANE S, YAMAMOTO H, NANASAWA T AND HIROTA T. (1989). Prognostic contributions of *Helix pomatia* and carcinoembryonic antigen staining using histochemical techniques in breast carcinoma. *Jpn J. Clin. Oncol.*, **19**, 127–134.

FUKUTOMI T, HIROHASHI S, TSUDA H, NANASAWA T, YAMAMOTO H, ITABASHI M AND SHIMOSATO Y. (1991). The prognostic value of tumor-associated carbohydrate structures correlated with gene amplifications in human breast carcinomas. *Jpn J. Surg.*, **21**, 499–507.

GANNON JV, GREAVES R, IGGO R AND LANE DP. (1990). Activating mutations in p53 produce a common conformational effect. A monoclonal antibody specific for the mutant form. *EMBO J.*, **9**, 1595–1602.

HAKOMORI S. (1989). Aberrant glycosylation in tumors and tumor-associated carbohydrate antigens. *Adv. Cancer Res.*, **52**, 257–331.

HALL PA, LEVINSON DA, WOODS AL, YU CC-W, KELLOCK DB, WATKINS JA, BARNES DM, GILLETT CE, CAMPLEJOHN R, DOVER R, WASEEM NH AND LANE DP. (1990). Proliferating cell nuclear antigen (PCNA) immunolocalization in paraffin sections: an index of cell proliferation with evidence of deregulated expression in some neoplasms. *J. Pathol.*, **162**, 285–294.

HIRAIZUMI S, TAKASAKI S, SHIROKI K, KOCHIBE N AND KOBATA A. (1990). Transfection with fragments of the adenovirus 12 gene induces tumorigenicity-associated alteration of N-linked sugar chains in rat cells. *Arch. Biochem. Biophys.*, **280**, 9–19.

HOLLSTEIN M, SIDRANSKY D, VOGELSTEIN B AND HARRIS CC. (1991). p53 mutations in human cancers. *Science*, **253**, 49–53.

JAPANESE RESEARCH SOCIETY FOR GASTRIC CANCER. (1981). The general rules for the gastric cancer study in surgery and pathology. I. Clinical classification. II. Histological classification of gastric cancer. *Jpn J. Surg.*, **11**, 127–139, 140–145.

JAPANESE RESEARCH SOCIETY FOR GASTRIC CANCER. (1993). *The General Rules for Gastric Cancer Study*, 12th edn, pp. 30–31. Kanehara: Tokyo (in Japanese).

JOYPAUL BV, HOPWOOD D, NEWMAN EL, QURESHI S, GRANT A, OGSTON SA, LANE DP AND CUSCHIERI A. (1994). The prognostic significance of the accumulation of p53 tumour-suppressor gene protein in gastric adenocarcinoma. *Br. J. Cancer*, **69**, 943–946.

KAKEJI Y, TSUJITANI S, MORI M, MAEHARA Y AND SUGIMACHI K. (1991*a*). Helix pomatia agglutinin binding activity is a predictor of survival time for patients with gastric cancer. *Cancer*, **68**, 2438–2442.

KAKEJI Y, KORENAGA D, TSUJITANI S, HARAGUCHI M, MAEHARA Y AND SUGIMACHI K. (1991*b*). Predictive value of Ki-67 and argyrolphilic nucleolar organizer region staining for lymph node metastasis in gastric cancer. *Cancer Res.*, **51**, 3503–3506.

KAKEJI Y, KORENAGA D, TSUJITANI S, BABA H, ANAI H, MAEHARA Y AND SUGIMACHI K. (1993). Gastric cancer with p53 overexpression has high potential for metastasising to lymph nodes. *Br. J. Cancer*, **67**, 589–593.

KAWAI T, SUZUKI M, TORIKATA C AND SUZUKI Y. (1991). Expression of blood group-related antigens and *Helix pomatia* agglutinin in malignant pleural mesothelioma and pulmonary adenocarcinoma. *Hum. Pathol.*, **22**, 118–124.

KIKUCHI-YANOSHITA R, KONISHI M, ITO S, SEKI M, TANAKA K, MAEDA Y, IINO H, FUKAYAMA M, KOIKE M, MORI T, SAKURABA H, FUKUNARI H, IWAMA T AND MIYAKI M. (1992). Genetic changes of both p53 alleles associated with the conversion from colorectal adenoma to early carcinoma in familial adenomatous polyposis and non-familial adenomatous polyposis patients. *Cancer Res.*, **52**, 3965–3971.

LAMPE BV, STALLMACH A AND RIECKEN EO. (1993). Altered glycosylation of integrin adhesion molecules in colorectal cancer cells and decreased adhesion to the extracellular matrix. *Gut*, **34**, 829–836.

LANE DP. (1992). p53, guardian of the genome. *Nature*, **358**, 15–16.

LIVINGSTONE LR, WHITE A, SPROUSE J, LIVANOS E, JACKS T AND TLSKY TD. (1992). Altered cell cycle arrest and gene amplification potential accompany loss of wild-type p53. *Cell*, **70**, 923–935.

MAEHARA Y, KOHNOE S AND SUGIMACHI K. (1990). Chemosensitivity test for carcinoma of digestive organs. *Semin. Surg. Oncol.*, **6**, 42–47.

MAEHARA Y, SAKAGUCHI Y, MORIGUCHI S, ORITA H, KORENAGA D, KOHNOE S AND SUGIMACHI K. (1992). Signet ring cell carcinoma of the stomach. *Cancer*, **69**, 1645–1650.

MORI M, KAKEJI Y, ADACHI Y, MORIGUCHI S, MAEHARA Y, SUGIMACHI K, JESSUP JM, CHEN LB AND STEELE Jr GD. (1993). The prognostic significance of proliferating cell nuclear antigen in clinical gastric cancer. *Surgery*, **113**, 683–690.

OLDEN K. (1993). Adhesion molecules and inhibitors of glycosylation in cancer. *Cancer Biol.*, **4**, 269–276.

PIERCE M AND ARANGO J. (1986). Rous sarcoma virus-transformed baby hamster kidney cells express higher levels of asparagine-linked tri- and tetraantennary glycopeptides containing [GlcNAc-β(1,6)Man α(1,6)Man] and poly-*N*-acetyllactosamine sequences than baby hamster kidney cells. *J. Biol. Chem.*, **261**, 10772–10777.

SCHUMACHER U, KRETZSCHMAR H, BROOKS S AND LEATHEM A. (1992). *Helix pomatia* lectin binding pattern of brain metastases originating from breast cancers. *Pathol. Res. Pract.*, **188**, 284–286.

SELL S. (1990). Cancer-associated carbohydrates identified by monoclonal antibodies. *Hum. Pathol.*, **21**, 1003–1009.

THOMAS M, NOGUCHI M, FONSECA L, KITAGAWA H, KINOSHITA K AND MIYAZAKI I. (1993). Prognostic significance of *Helix pomatia* lectin and c-*erb*B-2 oncoprotein in human breast cancer. *Br. J. Cancer*, **68**, 621–626.

UCHINO S, TSUDA H, MARUYAMA K, KINOSHITA T, SASAKO M, SAITO T, KOBAYASHI M AND HIROHASHI S. (1993). Overexpression of c-*erb*B-2 protein in gastric cancer. *Cancer*, **72**, 3179–3184.

VOGELSTEIN B AND KINZLER KW. (1992). p53 function and dysfunction. *Cell*, **70**, 523–526.

WASEEM NH AND LANE DP. (1990). Monoclonal antibody analysis of the proliferating cell nuclear antigen (PCNA). Structural conservation and the detection of a nucleolar form. *J. Cell Sci.*, **96**, 121–129.

YIN Y, TANINSKY MA, BISCHOFF FZ, STRONG LC AND WAHL GM. (1992). Wild-type p53 restores cell cycle control and inhibits gene amplification in cells with mutant p53 alleles. *Cell*, **70**, 937–948.